# Hypochondriasis

**Published:** 1863-04

**Authors:** 


### Art. IX.—HYPOCHONDRIASIS.

(*Translated from the German.*†)

Romberg designates hypochondriacs "virtuosos on the sensitive nerves," though this virtuosoship they are only able to exercise in a painful manner. Their feelings refer wholly to their health, the condition of which lays claim to all their attention, and by which morbid feelings are produced in the organism either through vividly intense conceptions, or sensations occurring in the organism are interpreted erroneously.

These morbid sensations, which may be produced centrally by conceptions, the hypochondriac may, to a certain extent, transfer wherever he will, as according to the idea he adopts he turns his attention to this or that part of the body.

Even, however, in the cases where an actual sensation, arising peripherically, does not produce a corresponding conception, the cause of this imperfect perception must be transferred to the brain. From these considerations, as well as that hypochondriasis may be produced by the perusal of medical writings, through intercourse with hypochondriacs, through fear and anxiety during epidemics, or after an individual has exposed himself to contagious diseases, it follows that hypochondriasis may arise from peripherical or central causes, may depend upon a morbid condition of the brain, and that even in those cases where, with the most careful investigation, no organic disease

---

† *Pathology and Therapeutics of Mental Diseases.* By Dr. Maximilian Leidesdorf.

can be proved, we are not justified in holding the sufferings of the hypochondriac as imaginary, his complaints as groundless and petulant.

The excited morbid feeling, the different hallucinations of sensation, are not products simply of the imagination, because one free from hypochondriasis is not generally capable of producing by the power of his will and imagination similarly intense symptoms.

The fact that in the most hypochondriacal, stomachic symptoms generally appear, weakens in no degree the above expressed opinion. We consider only from what we know, that the first disorders therein referred to are of such a nature that they are produced under the influence of the nervous system. These symptoms are generally a feeling of pressure and fulness in the stomach, combined with the chemical anomalies of flatulence and heartburn, and with this is associated deficient appetite, which sometimes alternates with voracity.

Flatulency and constipation are generally symptoms, and increase the psychical condition. The raised diaphragm narrows the space of the thorax, the respiration is difficult, palpitation makes its appearance; with these are associated lightness of the head and headache, and even the muscular movements are tremulous. That there is no want of abnormal sensations is easily imagined. These extend regularly from the lower part of the abdomen to the thorax, and tend most frequently to that place to which the patient principally directs his attention.

All the original symptoms that we observe in hypochondriacs point to the psychical origin of the disease, whose position can only be in the brain: it may always make its appearance along with some kind of pathological condition, or refer itself to disease of the brain as its starting-point.

The frequently occurring diseases of the liver and spleen are in and by themselves never to be considered as a direct cause of hypochondriasis; whilst, however, the decidedly chronic affections of this kind interfere with the nutriment in general, and deteriorate the elaboration of the blood, the brain will be injured in its normal nutrient relations and its functions.

All chronic diseases, generally the most painful and incurable, will easily produce a persistently depressed and mournful tendency, still that is not hypochondriasis; the characteristic of which is in no case to be sought for in its origin in diseases of the abdomen, as has been done without foundation, because the slightest or the most intense diseases and changes in the abdomen may take place without hypochondriacal symptoms.

From whatever kind of disturbance of the digestive organs we may derive the origin of hypochondriasis, it only exists when the irresistible tendency prevails to call forth by mental concentration new abnormal sensations, and where false judgments and hallucinations relate exclusively to the health of the patient.

The central influence may be long excited under such circumstances, concerning sensations in any part of the body; but the influence of the concentrated attention dwelling, however little, upon the predominant condition, will at last appear very clearly. In reference to this I remember, amongst other similar cases, one enumerated by Chomel.

A physician at Lyons had been present at the examination of several persons who died of hydrophobia, and was in consequence of this seized with the idea that the poison had inoculated him. All attempts at drinking produced cramp of the stomach and choking. He passed several days in desponding anxiety, until his friends succeeded in convincing him that his disease existed only in his imagination. The powerful action of an emotion points out clearly the influence of the brain upon the predominating condition; and it is not going too far to say, that under the influence of the hypochondriacal disposition the nutrient relations will after a time, and gradually, be destroyed, and that these disturbances may proceed to the most palpable structural changes. In particular, its particular mode of origin points to the psychical nature of the disease—to its position in the brain.

It is the disposition of the patients which begins to change, without external motives; they become sensitive, peevish, disheartened; a mistrustful, irritable temper attacks them; an anxiety, proceeding to the highest degree, seizes them; painful sensations appear in the different regions of the sensitive nerves; and whilst the attention of the patient concentrates itself upon these sensations, he feels he himself causes them to terminate in very severe diseases, by which he is punished, and whose position and name may change as quickly as the sensations in which he believes to recognise them. When the hypochondriac is occupied with these sensations and the sensations corresponding to his depressed condition, he is at the same time occupied incessantly in abundant explanations of them, and seeks in every way to be free of them; and it is known how such patients seek advice not only from different physicians, but also in medical books wholly misunderstood by them.

Thus is laid in this alone an important differential characteristic between hypochondriasis and melancholia. The hypochondriac seeks above all, advice and help; he easily agrees with others, and has confidence, though wavering and of short duration. The melancholic seeks no help, except in plans, or in the consummation of suicide; every other he puts aside, and considers them as useless. In the hypochondriac is maintained, in spite of the depressed condition of the mind, in spite of the abnormal sensations and perceptions connected with these, still presence of mind and a certain proportion of self-control with it. For, setting aside the ideas concerning his organism, the hypochondriac applies himself in his usual way to business, and fulfils his duties.

Only in the highest grades of hypochondriasis an actual loss of intelligence shows itself, in which the patient becomes nearly incapable of every mental activity. These advanced stages we frequently see passing into true melancholia and melancholic fatuity, in which generally appear to exist delusions widely extended and ramifying—that they are under secret influences, that they are magnetised, &c.

The course of hypochondriasis is generally chronic, being accompanied with remissions and intermissions; still, there are cases which take a more rapid course, and we generally see such instances arise during epidemic diseases under the influence of fear. The prognosis is the more favourable the more recent the disease, the freer the mind, the more sociable the patients, the less they are reduced, and so long as no organic disease has appeared, which, in another way, may prove dangerous or incurable.

The treatment of hypochondriasis demands before everything great patience and foresight on the part of the physician. We dare neither yield too much to the complaints of the patient, who would frequently be encouraged by this to make new ones, nor still less may he declare his sufferings as perfectly unimportant, by which we would alienate the confidence of the patient. A great part of that about which he complains he indeed actually feels. "The sensations of the patient," says Romberg, "are indeed imagined, but imagined by the mind as existing in the body. As to the sensation, it makes no difference whether the irritation exists at peripherical or central terminations of the nerve-fibre, whether it is produced through mental concentration, or through a mechanical, chemical, or organic cause."

We will not, therefore, be able to remedy his sufferings by an uncharitable judgment. By yielding to a certain extent to his complaints, as well as by an indispensably careful examination of his entire organism, we will very soon gain his confidence, and an opportunity will not be long wanting to obtain a certain mental superiority, which we must strive to maintain by firmness. Under such circumstances, it will be less difficult to act upon the judgment of the hypochondriac, not by direct contradiction, but because we give him a proof that in spite of fear, and of his mental and bodily depression, he may be able to act, may even become quite well. With this fundamental dogma in the treatment of hypochondriasis is to be ranked a second; the attention shall be turned aside from his bodily condition, and shall be led into a state artfully awakened and hitherto unknown to him. To this end recommend themselves travelling, exercise, driving, riding, swimming, gymnastics, cold and warm baths, sea-bathing, and mineral baths; I have also given, when not contra-indicated, emetics with good result.

How much that is best and most judicious, psychical derivation may effect in a particular case, must be deferred to the judgment of the physician, and a hint of the indication suffices here.

The existing and discovered disturbance and change in the organs forms another not less important point of attack for therapeutics. But the manifold troublesome sensations of these demand an amelioration which we may attempt by morphia, or in strong feelings of anxiety or palpitation by digitalis—even by quinine; in many cases also by the administration of cold, it may be by the drinking of cold water or by the sucking of pieces of ice. The diseases of the stomach we seek to remedy in pyrosis by magnesia or soda; excessive flatulency in the stomach and bowels by the use of ethereal oils, aromatics, by severer remedies, and by inunction of the balsam vitæ Hoffmanni. We treat the obstinate constipation of hypochondriacs by the use of clysters, jalap, aloes, rhubarb, senna, or these combined, in contradistinction to the habit of drinking mineral waters. Very few find the purgative salts a useful medicine, and in particular we avoid strong evacuants, which generally weaken the patient and injure his timid mind still more.

That in weak, anæmic individuals quinine, iron, sea-baths, &c., are indicated, hardly requires to be mentioned.

It is equally to be understood that during the whole course of treatment, we have to attend to the normal action of all the functions—digestion, the evacuations, the secretion of the skin, and particularly sleep. In a word we keep in view the powers of the constitution.

The efficacy of specific means acting directly upon the nervous system is in general very unsatisfactory, and we should never forget that the administration of a narcotic has frequently been followed by an unfavourable action.

We must, therefore, in the administration of these, be considerate and cautious. Patients who, in consequence of their own experiments, or at the advice of others, have employed many physicians, or undergone frequent treatment, we leave a certain time without medicine, and only prescribe under necessity, to satisfy their habit and demands, a harmless remedy. If there occurs, during the course of hypochondriasis, the development of any organic disease, the treatment of such an affection must be conducted according to its nature and site.

---

### Art. X.—ON THE DIAGNOSIS AND TREATMENT OF MORBID IMPULSE.

